# Integrative genomic meta-analysis reveals novel molecular insights into cystic fibrosis and ΔF508-CFTR rescue

**DOI:** 10.1038/s41598-020-76347-0

**Published:** 2020-11-25

**Authors:** Rachel A. Hodos, Matthew D. Strub, Shyam Ramachandran, Li Li, Paul B. McCray, Joel T. Dudley

**Affiliations:** 1 Mount Sinai School of Medicine, Institute for Next Generation Healthcare, New York, NY USA; 2 Courant Institute for Mathematical Sciences, New York University, New York, NY USA; 3 Department of Pediatrics, Carver College of Medicine, University of Iowa, Iowa City, IA USA; 4 Interdisciplinary Graduate Program in Genetics, University of Iowa, Iowa City, IA USA; 5 Present Address: BenevolentAI, Brooklyn, NY USA; 6 Present Address: Editas Medicine, Cambridge, MA USA; 7 Present Address: Sema4, Stamford, CT USA

**Keywords:** Bioinformatics, Gene expression analysis, Respiratory tract diseases

## Abstract

Cystic fibrosis (CF), caused by mutations to *CFTR*, leads to severe and progressive lung disease. The most common mutant, ΔF508-CFTR, undergoes proteasomal degradation, extinguishing its anion channel function. Numerous in vitro interventions have been identified to partially rescue ΔF508-CFTR function yet remain poorly understood. Improved understanding of both the altered state of CF cells and the mechanisms of existing rescue strategies could reveal novel therapeutic strategies. Toward this aim, we measured transcriptional profiles of established temperature, genetic, and chemical interventions that rescue ΔF508-CFTR and also re-analyzed public datasets characterizing transcription in human CF vs. non-CF samples from airway and whole blood. Meta-analysis yielded a core disease signature and two core rescue signatures. To interpret these through the lens of prior knowledge, we compiled a “CFTR Gene Set Library” from literature. The core disease signature revealed remarkably strong connections to genes with established effects on CFTR trafficking and function and suggested novel roles of EGR1 and SGK1 in the disease state. Our data also revealed an unexpected mechanistic link between several genetic rescue interventions and the unfolded protein response. Finally, we found that C18, an analog of the CFTR corrector compound Lumacaftor, induces almost no transcriptional perturbation despite its rescue activity.

## Introduction

Cystic fibrosis (CF) is the most common lethal genetic disease among individuals of European ancestry. It is caused by mutations to the cystic fibrosis transmembrane conductance regulator (*CFTR*) gene, which encodes an anion channel expressed in epithelia and other cell types. Loss of CFTR function leads to impaired chloride, bicarbonate, and liquid secretion, resulting in buildup of thick, sticky mucus and a progressive cycle of infection, inflammation, and tissue remodeling. The most common mutation, ΔF508, results in production of a misfolded CFTR protein that is degraded in the proteasome, preventing its trafficking to the plasma membrane. However, mutant proteins that bypass the cell’s quality control checkpoints and reach the plasma membrane retain some level of function^1^. Accordingly, a major focus of CF therapeutic research is to find ways to rescue the trafficking and display of ΔF508-CFTR at the plasma membrane, thereby restoring anion channel function. Recent success has proven the effectiveness of this approach, with FDA approvals of double- and triple-combination therapies that interact directly with ΔF508-CFTR to improve its folding stability and function^2,3^. While early results are promising, these treatments are costly and are not applicable to patients with all heterozygous mutations. Hence there is a continued need for new therapies.

Alternative strategies to rescue the trafficking and display of ΔF508-CFTR act through more indirect means such as by altering the activity or expression of machinery involved in CFTR folding, processing, and/or trafficking. Indeed, there are many established rescue interventions believed to act through such indirect means (e.g., low-temperature treatment^1^; overexpression of miR-138^4^; or miR-16^5^; or knockdown of *AHA1*^6^, *SIN3A*^4^, *SYVN1*^7^, or *NEDD8*^7^). However, these interventions have limited efficacy or are poorly understood. On the other hand, small-molecule therapeutics such as the FDA-approved VX-809 and its analog C18 likely interact directly with the mutant CFTR protein, with less known about their effects on the broader cellular environment^8,9^. Given the current limitations on mechanistic knowledge, a better understanding of how the cellular environment is altered in CF cells and is manipulated by various rescue strategies, could lead to the development of novel targets and improved therapies.

Here, we performed a meta-analysis of transcriptomic data from both original microarray experiments and public sources to accomplish two aims: (1) define gene expression changes present across various CF vs. non-CF comparisons; and (2) elucidate mechanisms of ΔF508-CFTR rescue. We studied four categories of experiments: one that compared CF vs. non-CF expression in relevant human tissues, and three types of in vitro rescue strategies: (1) low-temperature rescue; (2) RNAi-based rescue (namely, siRNA-mediated knockdown of *SYVN1*, *NEDD8*, or *SIN3A*, or overexpression of miR-138); and (3) chemical rescue via C18. Systematic comparison and meta-analysis of these datasets yielded a core signature of the CF disease phenotype and two core signatures associated with ΔF508-CFTR rescue. To study the core signatures through the lens of prior domain knowledge, we compiled a CFTR Gene Set Library comprising 60 gene sets with various associations to CF or CFTR. Using this and other resources, we developed an original bioinformatic workflow to analyze and interpret these signatures.

## Results

**Figure 1:**
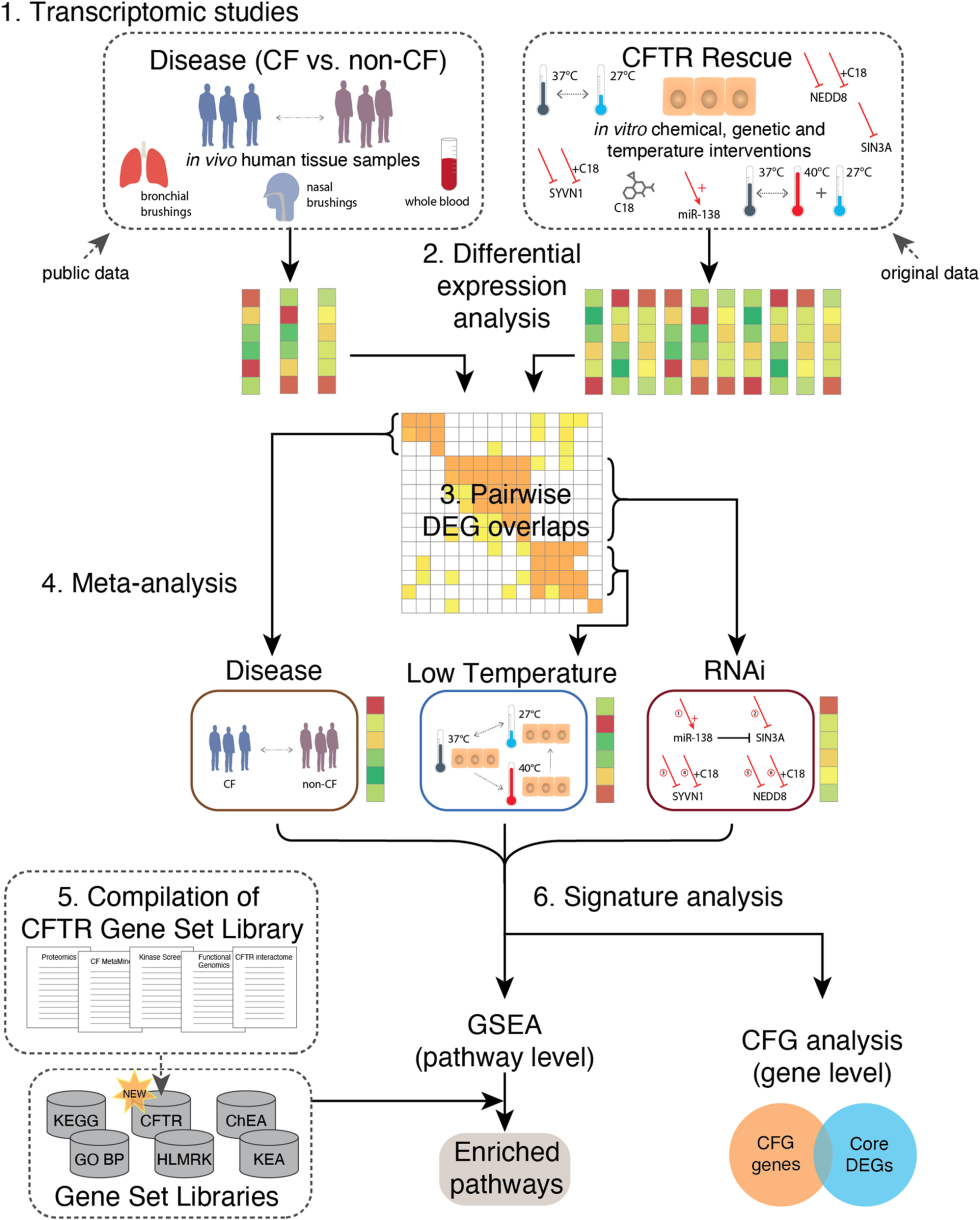
Overview of workflow. (1) Fourteen transcriptomic studies were generated and compiled, including three studies characterizing in vivo transcriptional alterations in CF vs. non-CF human subjects (left), and 11 in vitro rescue treatments (right). (2) Raw data were processed into 14 differential expression signatures using a standardized pipeline. (3) Pairwise comparison of DEGs extracted from these signatures led to (4) meta-analysis of three data subgroups, yielding core signatures of disease (*CF-Meta*), low temperature rescue (*LowTmp-Meta*), and RNAi-based rescue (*RNAi-Meta*). (5) Sixty gene sets associated with CF or CFTR were compiled from 34 publications into a CFTR Gene Set Library, which includes the CFTR functional genomics (CFG) gene set. (6) Each core signature was analyzed by Gene Set Enrichment Analysis (GSEA) and also compared to the CFG genes. *DEGs* differentially expressed genes, *HLMRK* Hallmark pathways.

An overview of the entire informatics pipeline is shown in Fig. 1. We first present differential expression analysis of the 14 compiled datasets and subsequent pairwise comparisons of the results.

### Differential expression analysis of 14 gene expression studies

**Figure 2:**
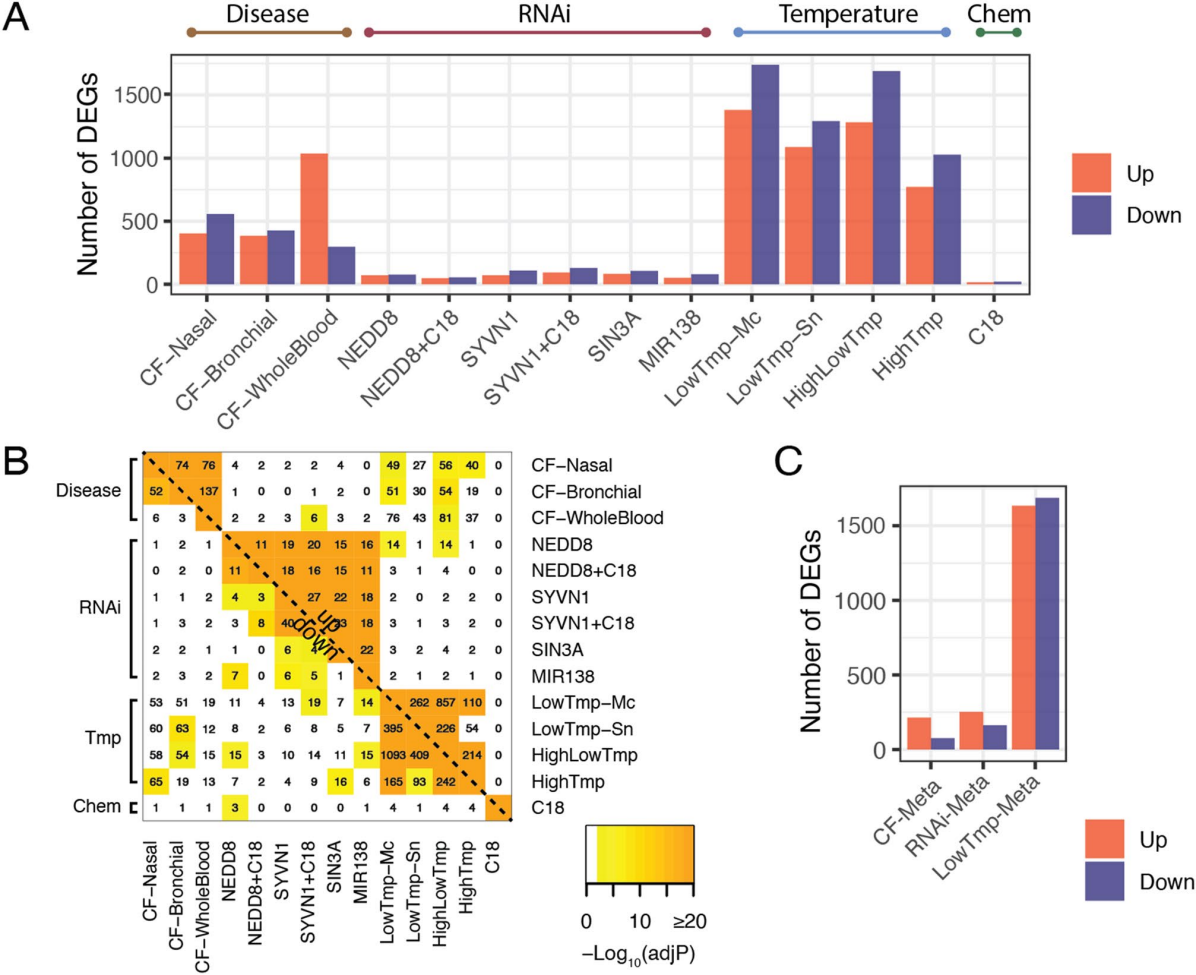
Systematic comparison and integration of 14 CF expression signatures into three meta-analyses. (**A**) Number of differentially expressed genes (DEGs) in 14 individual differential expression signatures. (**B**) Similarity matrix comparing pairwise DEG overlaps among each of the 14 signatures. The upper-right [lower-left] regions correspond to overlap of up- [down-] regulated gene sets. Non-white coloring indicates significance at a Benjamini–Hochberg adjusted *p* < 0.05, and the numbers correspond to intersecting DEGs. (**C**) Number of DEGs from the three core signatures resulting from meta-analysis. *Tmp* Temperature.

For this analysis, we used 14 gene expression datasets (see top of Fig. 1), of which ten were generated specifically for this study, and four were compiled from public sources. These datasets include two comparisons of CF vs. non-CF airway epithelia, one comparison of CF vs. non-CF whole blood, ten comparisons of rescue interventions in CFBE41o- cells vs. untreated controls, and one comparison of high-temperature treatment of CFBE41o- cells vs. a negative control. Standard data normalization and differential expression analysis were applied uniformly to each dataset to generate 14 lists of up- and down-regulated differentially expressed genes (DEGs); see “Methods” for details and Additional File 1 for gene lists. The number of DEGs identified per signature varied considerably (see Fig. 2A) but was generally consistent within an experimental category. More specifically, the temperature interventions yielded thousands of DEGs, the disease signatures yielded hundreds, and the genetic (RNAi) and chemical (C18) interventions yielded tens or hundreds.

#### C18 induces minimal transcriptional perturbation

Among the signatures analyzed, C18 treatment yielded the fewest DEGs (34 total, 14 up- and 20 down-regulated), suggesting that the compound induces a relatively small transcriptional perturbation in CFBE41o- cells. We also studied the transcriptional impact of C18 when combined with knockdown of either *NEDD8* or *SYVN1*. We found that the *NEDD8* + *C18* signature was highly correlated (0.89) with the *NEDD8*-alone signature, and that DEGs overlapped significantly between the two signatures (*p* < 1e − 17). Likewise, the *SYVN1* + *C18* signature was highly correlated (0.83) with that of the isolated *SYVN1* knockdown, and again DEGs overlapped significantly (*p* < 1e − 49). Hence, we conclude that C18 did not substantially change the transcriptional state in any of these three experiments.

### Comparative analysis of disease and rescue signatures

To determine whether it would be reasonable to perform a meta-analysis on these heterogeneous data, we identified the intersection of DEGs between pairs of signatures (see step 3 in Fig. 1). We reasoned that the signatures should only be combined in a meta-analysis if they exhibit significant similarity at the level of differential expression. Fisher’s exact tests were used to evaluate the significance of the number of DEGs in each pairwise overlap (adjusted using Benjamini-Hochberg^10^). The results are shown in Fig. 2B, and the many significant similarities are detailed below.

#### Transcriptional alterations in CF are concordant across airway and whole blood

Comparisons of disease signatures revealed significant overlaps in four of six cases (*p* < 1e − 16, see Fig. 2B), including all three comparisons of up-regulated gene sets. Down-regulated genes were significantly concordant between the nasal and bronchial brushings, but not significant when comparing these airway studies to those down-regulated in whole blood.

#### Multiple temperature and genetic interventions induce consistent expression perturbations

Also shown in Fig. 2B are comparisons among the various rescue signatures. In addition to the expected agreement^11^ among multiple low-temperature signatures, we observed high concordance among the six RNAi-based signatures, especially among up-regulated gene sets. Several comparisons between RNAi and low-temperature interventions, and between disease and rescue signatures, were also significant, as shown in Fig. 2B.

#### C18 has low correspondence to other rescue interventions

We compared the C18 (-only) signature with the other rescue signatures, testing for genome-wide correlations and overlap of DEGs. All correlations were < 0.10, and although one DEG comparison was significant (with down-regulated genes from the *NEDD8* intervention, *p* < 1e − 6), this involved only three genes (*SPAG4*, *PPFIA4*, and *LOC642121*; Fig. 2B).

### Overview of meta-analysis

Based on these analyses, we excluded the *C18* signature from the meta-analysis due to its lack of concordance with the other signatures, and also excluded the *HighTmp* signature due to its known lack of a rescue phenotype. We split the remaining signatures into three groups: (1) the three disease signatures (2) the six RNAi signatures, and (3) the three low-temperature signatures (center of Fig. 1). For each of these three groups we performed a meta-analysis. Specifically, we applied the variance pooling approach, in which logFC estimates for each gene are combined across studies (with weights inversely proportional to their gene-specific SEM) into a unified logFC estimate and corresponding SEM. This approach ensures that studies with more uncertainty regarding an individual gene will have less effect on the final estimate. See “Methods” for details and Additional File 2 for output.

The three resulting “core” signatures (subsequently referred to as the *CF-Meta, LowTmp-Meta*, and *RNAi-Meta* signatures, respectively) yielded varying numbers of DEGs, shown in Fig. 2C. Comparing with the corresponding input signatures in Fig. 2A, we found that the *CF-Meta* signature had somewhat fewer DEGs relative to its inputs while both the *LowTmp-Meta* and *RNAi-Meta* signatures had an increase in DEGs relative to their inputs. Note that unless otherwise specified, these core signatures will refer to the logFC estimates for all measured genes in these analyses, as opposed to the restricted set of DEGs.

In subsequent sections, after describing the compilation and analysis of the CFTR Gene Set Library, we present detailed analysis of each core signature as illustrated in the bottom half of Fig. 1.

### Compilation and analysis of the CFTR gene set library

To study the core signatures through the lens of prior domain knowledge, 60 gene sets with associations to CF or CFTR were compiled from 34 publications. Sources included studies in proteomics, functional genomics, transcriptomics, and expert-curated pathways. Here, we describe the three sources highlighted in subsequent results; details of all 60 gene sets comprising the CFTR Gene Set Library are included in Additional File 3A and 3B. First, genes whose products are in the CFTR protein interactome, i.e., that physically interact with CFTR (either WT or ΔF508), were retrieved from Pankow, Bamberger^12^. This set includes several gene lists: the full “core” interactome, i.e., the set of 624 high-confidence interactors as defined by Pankow et al., as well as a number of subsets based on various functional categorizations such as folding and endocytosis. Second are a set of nine expert-curated pathways related to various aspects of CFTR processing, trafficking, and degradation from the CF MetaMiner platform^13^. Third, we compiled the results of functional genomics studies described in 25 publications. In these studies, a total of 6188 unique genes were individually either knocked down or overexpressed, and subsequent CFTR surface expression (either WT or ΔF508) or downstream channel function was measured. Among those tested were 294 genes with significant effects on CFTR (referred herein as the *CFTR Functional Genomics*, or *CFG*, gene set), broken further into *CFG*^**+**^, which are the 77 genes (1.2%) with a net positive effect (i.e., over-expression yielded rescue or knockdown led to reduced trafficking or function) and *CFG*^***−***^*,* the 236 genes (3.8%) with a net negative effect (i.e., over-expression led to reduced function or knockdown led to rescue). Several genes showed both positive and negative effects in different experiments and were included in both lists. We place particular emphasis on the CFG genes in later sections due to the direct experimental evidence linking them to CFTR trafficking and function. We start with an enrichment analysis of the CFG genes themselves, and in later sections compare these enrichments to terms enriched in the core transcriptional signatures.

#### Enrichment analysis of CFTR Functional Genomics genes highlights shared biology among genes affecting CFTR

To study the biology represented in the CFG genes, we performed hypergeometric enrichment analysis, revealing 232 gene sets that were over-represented relative to the 6188 gene background. Reassuringly, we observed strong enrichments for genes whose products are in the CFTR protein interactome. We also observed expected enrichments related to protein folding, quality control and degradation, including various terms related to the unfolded protein response (UPR), “ubiquitin-mediated proteolysis”, “ubiquitin cycle”, and CFTR-specific pathways such as “CFTR folding and maturation, normal and CF” and “Regulation of degradation of ΔF508-CFTR in CF”. In addition to these expected terms were enrichments related to mTOR signaling, autophagy, and endocytosis, as well as terms suggesting connections to a variety of kinases and transcription factors. See Additional File 4 for details. In later sections, we highlight some of the CFG genes and enrichments as they relate to the core signatures, but first we present an overview of the *CF-Meta* signature.

### Analysis of the *CF-Meta* signature

Of the 15,123 genes measured in at least two of the three CF studies, 290 (1.9%) were differentially expressed: 214 were up-regulated and 76 down-regulated. Each of the individual signatures was significantly positively correlated (*r* = 0.66–0.82, *p* < 1e − 15) with the *CF-Meta* signature, with the *CF-WholeBlood* signature most highly correlated. Among the most differentially expressed genes were many that are consistent with prior reports, providing internal validation of our meta-analysis approach. For example, the top ten most up-regulated genes (*GOS2*, *OSM*, *S100A12*, *PROK2*, *FGFBP1*, *BCL2A1*, *LILRB3*, *S100A9*, *IL1R2*, *ADM*) were also reported as such by either or both of Clarke et al.^14^ and Ogilvie et al.^15^ and are discussed in more detail therein.

#### Combined analysis of CF-meta and CFG genes suggests novel roles of EGR1 and SGK1 in CF biology

**Figure 3:**
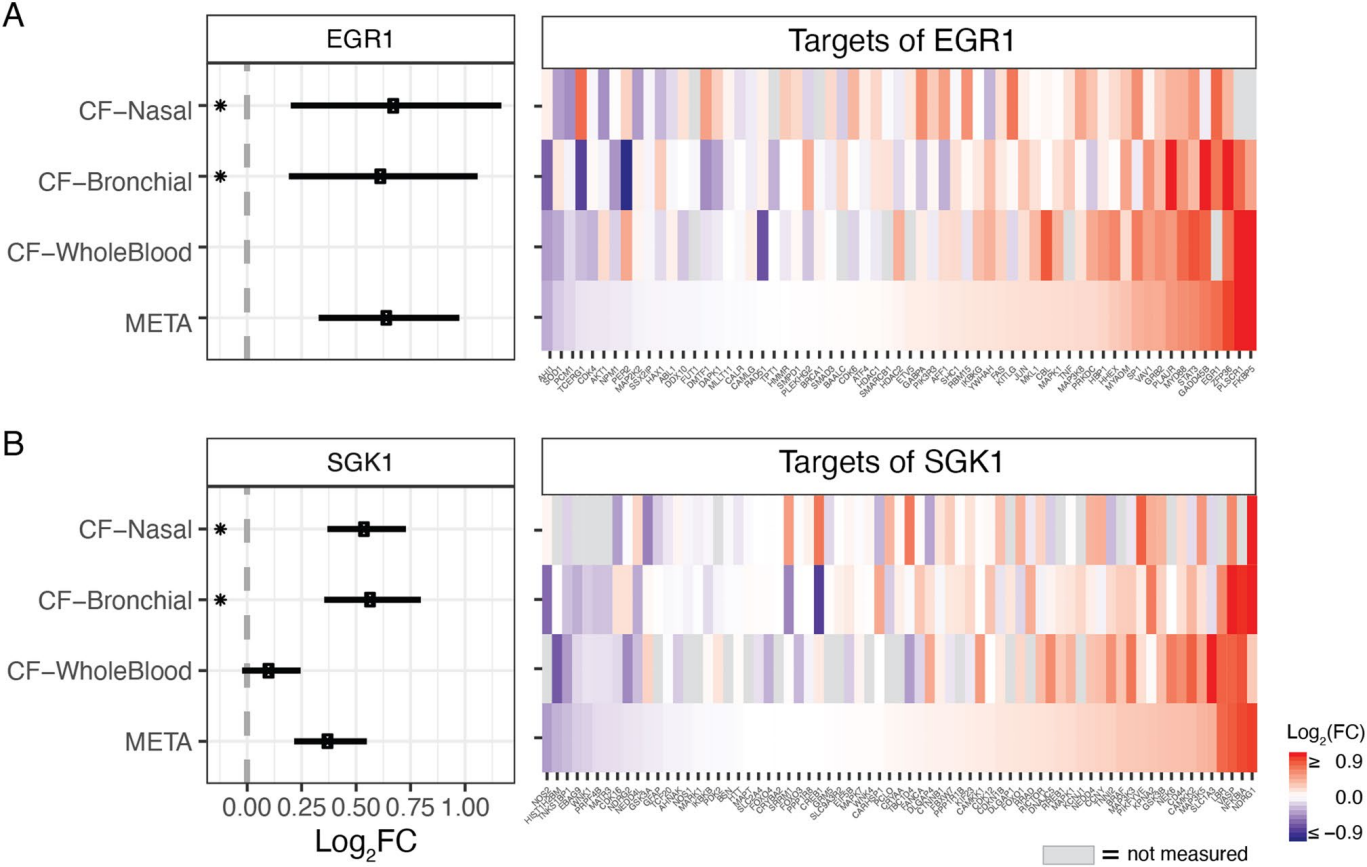
Differential expression of *EGR1*, *SGK1* and their downstream targets in CF vs. non-CF comparisons. (**A**) Left: Forest plot showing differential expression of *EGR1* transcript. Right: Differential expression heatmap of the regulatory targets of EGR1, according to the “EGR1-19374776 (human)” gene set from the ChEA gene set library. (**B**) Left: Forest plot showing differential expression of *SGK1* transcript. Right: Differential expression heatmap of the kinase targets of SGK1 (according to the “SGK1” gene set from KEA). In both heatmaps, columns indicate genes and are sorted according to the FC estimate from the meta-analysis. All units are log_2_(FC), (*): *p* < 0.01, and error bars indicate ± 1 SEM.

In addition to these expected results, we identified two genes that have not been previously reported in CF vs. non-CF comparisons and which may provide new insights into the CF disease state. The first is *EGR1* whose protein product is the early growth response 1 transcription factor. We observed three sources of evidence in our data linking EGR1 to CF or CFTR, namely: (1) up-regulation of the *EGR1* transcript in both the CF bronchial and nasal signatures (see Fig. 3A); (2) up-regulation of its transcriptional targets via ChIP-X Enrichment Analysis of the *CF-Meta* signature (see Fig. 3A and Table 1); and (3) over-representation of its transcriptional targets in the CFG genes (see Additional File 4). Second, we highlight *SGK1*, encoding the serum glucocorticoid-regulated kinase 1, which presented a similar set of evidence linking it to CF or CFTR: (1) its transcript was up-regulated in both the CF bronchial and nasal signatures (see Fig. 3B); (2) its kinase substrates were significantly up-regulated in a Kinase Enrichment Analysis^16^ of the *CF-Meta* signature (see Fig. 3B and Table 1); (3) its kinase substrates were significantly over-represented among the CFG genes (see Additional File 4); (4) SGK1 itself is a member of the CFG set based on the results of Caohuy et al.^17^, discussed further in the "discussion" section.

**Table 1:** Selected terms from enrichment analysis of the *CF-Meta* signature.

Term	ES	AdjP	DEG enrich	Gene set library	Enriched in CFG
TNFa signaling via NFkB	0.75	0.0E + 00	7.41	HALLMARK	No
IL6 JAK STAT3 signaling	0.73	0.0E + 00	8.58	HALLMARK	No
Toll like receptor signaling	0.69	0.0E + 00	6.67	KEGG	Yes
Nod like receptor signaling	0.67	0.0E + 00	3.54	KEGG	No
TF substrates of EGR1	0.63	0.0E + 00	4.21	ChEA	Yes
Apoptosis	0.61	0.0E + 00	4.45	KEGG	No
Interferon gamma response	0.61	0.0E + 00	3.48	HALLMARK	No
mTOR signaling pathway	0.52	2.6E − 02	3.19	KEGG	Yes
Kinase substrates of SGK1	0.52	5.1E − 03	2.27	KEA	Yes
Regulation of MAP kinase Activity	0.50	9.5E − 03	3.42	GO_BP	No
Endocytosis	0.50	0.0E + 00	1.52	KEGG	Yes
TGF beta signaling	0.49	2.6E − 02	2.01	HALLMARK	No
Autophagy	0.37	5.1E − 03	2.26	CF	Yes

*ES* enrichment score, *AdjP* adjusted *p*-value, *DEG Enrich* fold enrichment, denoting the ratio of the number of *CF-Meta* DEGs observed vs. expected within the gene set, *Enriched in CFG* whether the term was also significant in the CFG enrichment analysis.

#### CF-Meta signature is enriched for immune and MAPK signaling and apoptosis

GSEA revealed 242 gene sets out of 1710 tested that were significantly enriched; 240 of them (99%) had positive scores indicating up-regulation. As expected, the enriched terms included many signaling pathways related to immune or inflammatory responses, such as “IL6 JAK STAT3 signaling”, “TNFα signaling via NF-κB”, “Toll-like receptor signaling”, “Nod-like receptor signaling”, and “Interferon gamma response”, many of which were mentioned by Ideozu et al.^18^. Other enrichments suggested increased levels of TGFβ signaling, MAPK signaling, and apoptosis. See Table 1 for selected enrichments and Additional File 4 for the complete list.

#### CF-Meta enrichments significantly overlap with those from CFTR Functional Genomics set

Directly comparing the *CF-Meta* DEGs with the CFG set revealed nine genes in common (*ALOX5AP*, *BIN2*, *DYDC2*, *FGFBP1*, *MAPK3*, *PIK3CD*, *QPCT*, *RAF1*, *S100A8*), however this overlap was not significant. Remarkably however, 79 of the *CF-Meta* enriched gene sets were also enriched in the CFG analysis, which was highly significant (Fisher’s exact *p* < 1e − 18). Common terms, in addition to the SGK1 and EGR1 gene sets mentioned above, included targets of other kinases and transcription factors, several terms related to mTOR signaling, as well as endocytosis and autophagy. See the “CFG” tab of Additional File 4 for details*.*

### Analysis of the *LowTmp-Meta* signature

Next, we analyzed the signature characterizing low-temperature treatment of CFBE41o- cells. Of the 32,285 genes examined, 3321 (10.3%) were differentially expressed in the meta-analysis (1634 up; 1687 down), highlighting the profound transcriptional impact of low-temperature treatment on CFBE41o- cells, consistent with previous work^11^. Each of the individual low-temperature signatures was more highly correlated with the core signature than with the other individual signatures.

#### Low-temperature up-regulates immune and inflammatory pathways and down-regulates metabolic function

GSEA revealed 67 gene sets out of 1710 tested that were significantly enriched in the *LowTmp-Meta* signature; 28 were up- regulated and 39 down-regulated. We observed a broad pattern of up-regulation of immune and inflammatory responses (including “TNFα signaling via NF-κB”, “interferon alpha response”, “interferon gamma response”, and “IL6 JAK STAT3 signaling”), as well as broad down-regulation of cellular metabolism (including “fatty acid metabolism”, “galactose metabolism”, “propanoate metabolism”, “glycolysis gluconeogenesis”, and “valine, leucine, and isoleucine degradation”).

#### Low-temperature treatment regulates genes and pathways related to protein folding and trafficking

**Figure 4:**
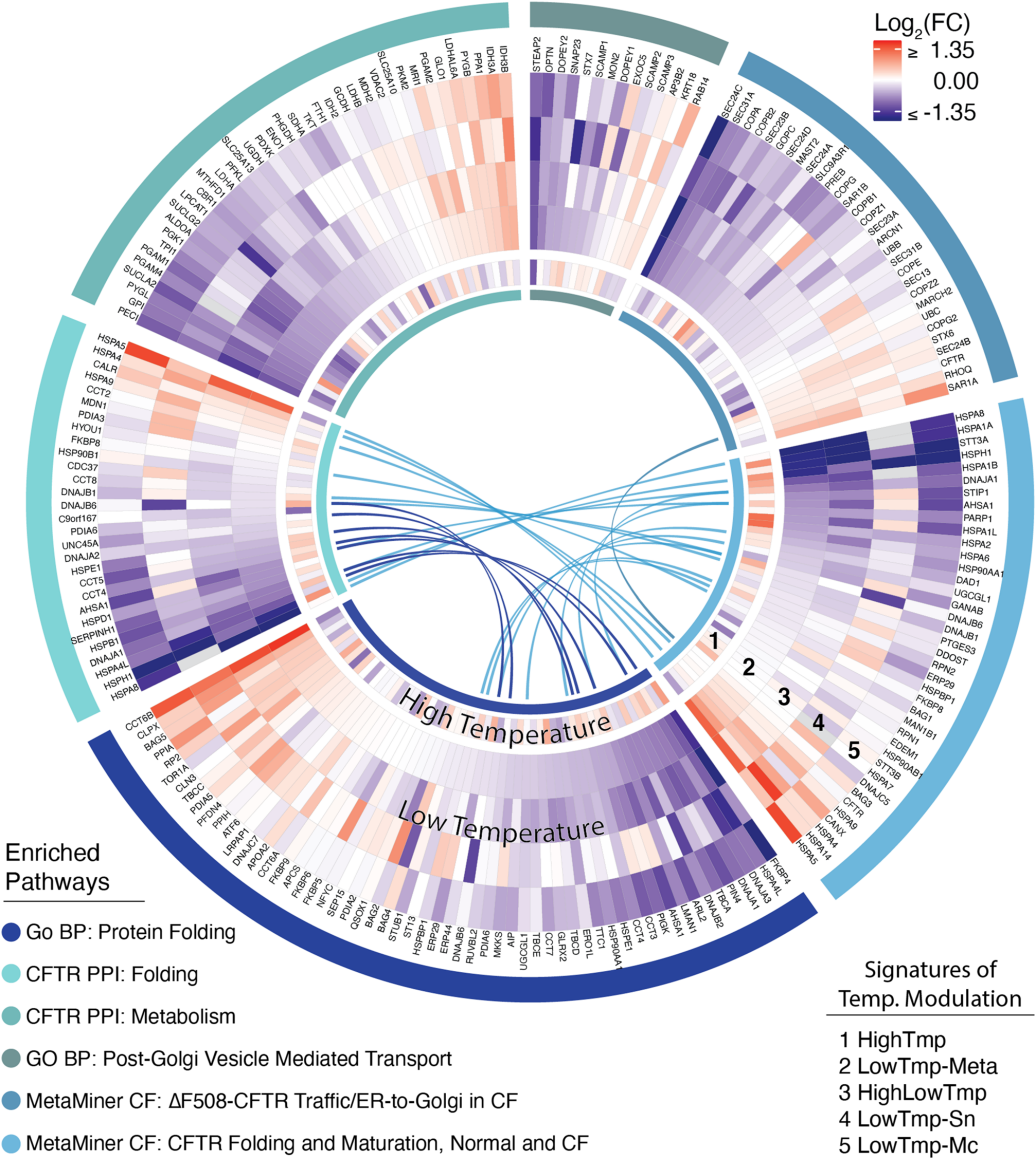
Temperature-induced modulation of selected CFTR-relevant gene sets. “Columns” emanating from the center represent genes, which are grouped according to pathways, as indicated by the colors in the outermost circle. Concentric circles represent individual signatures and/or the core *LowTmp-Meta* signature, as indicated by the numbered legend. Arcs in the center connect genes that are shared between pathways.

Consistent with prior observations that low-temperature incubation has broad effects on protein folding and trafficking, we identified several such pathways that were significantly enriched (see Fig. 4 and Additional File 4 for details). The most up-regulated term in the enrichment analysis (ES 0.88) was “PERK-mediated UPR”. Two additional pathways were down-regulated: “protein folding” and “post-Golgi vesicle-mediated transport.” Two CF MetaMiner pathways related to folding and trafficking were also significantly down-regulated: “CFTR folding and maturation, normal and CF” and “ΔF508-CFTR traffic/ER-to-Golgi in CF”. The former enrichment was driven by reduced expression of many chaperone proteins, and the latter by down-regulation of multiple genes encoding components of the COPII complex (see Fig. 4).

#### Low-temperature incubation down-regulates genes in the CFTR interactome

In addition, the “CFTR core interactome” identified by^12^ (see “Methods”) was significantly down-regulated. This was particularly true (i.e., enrichment scores were higher) for genes associated with either folding or metabolism (see Fig. 4 and Additional File 4). However, the metabolism-related subset of the CFTR interactome was also down-regulated to a similar degree in the *HighTmp* signature.

#### Intersection of LowTmp-Meta DEGs with CFG set reveals candidate mediators of rescue

Intersection of the DEGs from the *LowTmp-Meta* signature with the CFG set yielded 32 genes with a directionality that supports them as candidate mediators of low-temperature induced rescue of ΔF508-CFTR’s trafficking or function. Table 2 shows the 17 genes with |logFC| > log_2_(1.5). These include nine genes in CFG^**+**^ that were up-regulated in *LowTmp-Meta*, and 23 genes in CFG^***−***^ that were down-regulated. Intersection of the *LowTmp-Meta* and CFG enrichments revealed 12 gene sets, several of which are already mentioned above; however, this comparison was not significant. See Additional File 4 for details.

**Table 2:** Transcriptomic and functional genomic support for candidate mediators of low-temperature rescue of ΔF508-CFTR trafficking.

Symbol	Transcriptomics (*LowTmp-Meta*)			Functional Genomics
	LogFC ± SEM	AdjP	Direction	CFG
PSMD9	1.21 ± 0.47	2.7E − 2	Up	Positive^19^
NDUFAF5	1.15 ± 0.10	0.0E − 0	Up	Positive^20^
COX4NB	0.91 ± 0.16	2.0E − 4	Up	Positive^21^
EDN1	0.87 ± 0.34	2.8E − 2	up	positive^20^
AKAP8	0.85 ± 0.14	2.0E − 4	Up	Positive^22^
DNAJB2	− 0.63 ± 0.14	2.1E − 4	Down	Negative^23^
UBAC2	− 0.63 ± 0.09	2.0E − 4	Down	Negative^21^
DNAJA1	− 0.68 ± 0.10	2.0E − 4	Down	Negative^23^
SEC22B	− 0.68 ± 0.07	0.0E − 0	Down	Negative^24^
MET	− 0.69 ± 0.15	2.0E − 4	Down	Negative^25^
PANK1	− 0.72 ± 0.15	2.0E − 4	Down	Negative^25^
DNAJA3	− 0.76 ± 0.11	2.0E − 4	Down	Negative^23^
GTSE1	− 0.79 ± 0.08	0.0E − 0	Down	Negative^22^
UBE4B	− 0.91 ± 0.14	2.0E − 4	Down	Negative^23^
HSPA1A	− 1.29 ± 0.16	2.0E − 4	Down	Negative^7^
HSPA8	− 1.48 ± 0.33	2.1E − 4	Down	Negative^7,23^
RNASEL	− 1.57 ± 0.25	2.0E − 4	Down	Negative^26^

Direction column shows the sign of differential expression in the *LowTmp-Meta* signature. The CFG column shows the sign of each gene’s net effect on CFTR trafficking based on the referenced study, i.e. positive indicates membership in the CFG^**+**^ set, and negative indicates membership in CFG^**−**^.

*LogFC* logarithm (base 2) of the fold change, *SEM* standard error of the mean, *AdjP* adjusted *p*-value, *CFG* CFTR Functional Genomics.

### Analysis of the *RNAi-Meta* signature

Of the 30,486 genes represented in the *RNAi-Meta* signature, 415 (1.4%) were identified as DEGs, of which 252 were up-regulated and 163 down-regulated. Correlations between the individual signatures and the core signature ranged between *r* = 0.70 and 0.77, suggesting that all signatures contributed relatively equally to the meta-analysis estimates. The *RNAi-Meta* signature had three significant enrichments, all indicating up-regulation via positive enrichment scores. These included “PERK-mediated UPR” (ES 0.90) and “TNFα signaling via NF-κB” (ES 0.55). The third gene set was from the CF MetaMiner pathway “Mechanisms of CFTR activation by S-nitroglutathione” (ES 0.58), driven mainly by up-regulation of *HSP70* genes as well as *IL8* and *TXNRD1*.

#### Comparison between RNAi-Meta signature and CFG genes reveals limited gene and pathway connections

Intersection of the *RNAi-Meta* DEGs with the CFG genes set yielded two genes- *SYVN1* and *DNAJA3*. The former is expected, as one of the targeted genes from the RNAi experiments, and the latter is a member of the Hsp40 family of heat shock proteins. Two of the three *RNAi-Meta* enrichments (relating to UPR, and CFTR activation via S-nitroglutathione) were also significant in the CFG enriched list.

### UPR pathways are enriched in low-temperature and multiple RNAi signatures

**Figure 5:**
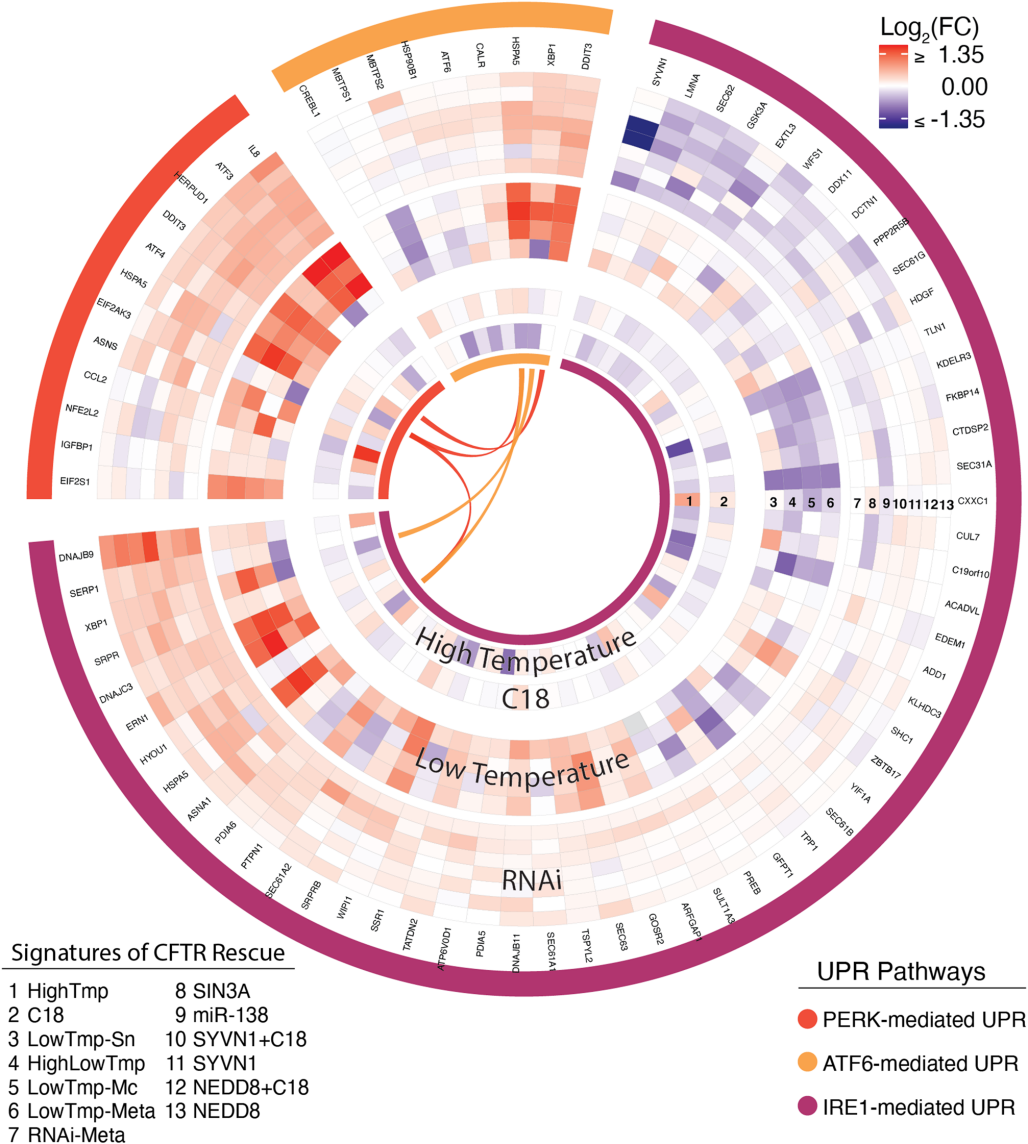
RNAi and low temperature, but not C18 or high temperature, induce consistent UPR transcriptional activation. “Columns” emanating from the center represent genes, which are grouped according to pathways, as indicated by the colors in the outermost circle. Concentric circles represent individual signatures and/or the core meta-analysis signatures, as indicated by the numbered legend. Arcs in the center connect genes that are shared between pathways.

The most up-regulated term in both the *LowTmp-Meta* and *RNAi-Meta* signature was “PERK-mediated UPR,” along with the Hallmark “UPR” pathway. Enrichment analysis of the individual RNAi-based signatures also revealed multiple significant UPR-related enrichments. In particular, “PERK-mediated UPR” was the first- or second-most enriched term in both *NEDD8* signatures (± C18) as well as in *SYVN1* + *C18* and *MIR138*, and “ATF6-mediated UPR” was the most enriched term in the *NEDD8* signature. Studying the expression patterns in UPR-related pathways induced by these interventions (see Fig. 5) revealed similar activation patterns induced by all of the genetic and temperature rescue signatures that were not significant in the C18 or high-temperature signatures. In the figure, the color coding indicates three gene sets based on the three canonical “arms” of the UPR: PERK-, ATF6-, and IRE1-mediated.

## Discussion

In this work, we elucidated transcriptional mechanisms underlying CF pathophysiology and known rescue interventions, with an emphasis on those that may affect CFTR processing and trafficking. We detected minimal transcriptional perturbation in cells treated with the CFTR corrector compound C18. In addition, we identified concordant transcriptional patterns across disease signatures, temperature interventions, and perhaps most surprisingly, RNAi interventions.

### Findings from C18 signature

Three distinct microarray experiments (comparing C18 vs DMSO, *NEDD8* knockdown, or *SYVN1* knockdown) revealed that C18 is transcriptionally “quiet”, affecting the expression of only a handful of genes. This suggests that: (1) C18’s rescue mechanism is unlikely to involve transcription, consistent with prior beliefs regarding its direct targeting of CFTR protein; and (2) the downstream (24-h) transcriptional impact of restoring ΔF508-CFTR function is likely to be minimal. Furthermore, the low concordance between the C18 signature and the other rescue signatures, along with the high concordance among other rescue signatures, suggests that C18 acts via a different mechanism, again consistent with our prior expectations. Because VX-809 is a chemical analog of C18 and is believed to act through similar mechanisms, we speculate that this molecule also induces little to no transcriptional effect. However, although C18 appears to be transcriptionally “quiet”, we cannot exclude the possibility that it exerts transcriptional effects at other timepoints, or that the small number of DEGs identified at the 24-h timepoint are biologically important.

### Findings from the *CF-Meta* signature

The meta-analysis of three datasets characterizing CF vs. non-CF airway epithelia and whole blood revealed consistent expression alterations that transcend tissue-specific differences. These results served as an important internal control and validation of the analysis strategy. We observed a striking overlap among enriched terms between the *CF-Meta* and CFG enrichments, supporting the validity of the *CF-Meta* signature. This overlap also suggests that while transcriptional alterations in CF cells may include responses to downstream immune signaling resulting from CF pathophysiology, they may also be linked to the core defect in CF, related to CFTR protein processing and trafficking. The vast majority of *CF-Meta* enriched gene sets had positive enrichment scores, suggesting a general increase in the activity of CF cells in response to the mutant protein or downstream inflammation.

We identified two genes, *EGR1*, and *SGK1*, not previously reported in CF vs. non-CF comparisons, which may play a role in the disease state. Our analysis provided evidence of increased levels and activity of the EGR1 transcription factor and the SGK1 kinase in CF airway epithelia, as well as over-representation of their target genes in the CFG set. EGR1 is a transcription factor involved in inflammation, wound repair, and fibrosis in multiple tissues^27^. Recent work showed that *EGR1* mRNA expression in peripheral blood leukocytes is significantly higher (FC > 4.5) in CF patients with mild vs. severe lung disease^28^. *SGK1* transcript is upregulated in CF lungs^29^, and SGK1 (a serine/threonine kinase) positively regulates both the trafficking and channel activity of CFTR and other ion channels^17,30,31^. In particular, Caohuy et al.^17^ claim that SGK1 inhibits, via phosphorylation, the activity of NEDD4-2, an E3 ubiquitin ligase that interacts with CFTR and promotes its degradation^17,19^. Taken together, we speculate that elevated activity of one or both of these molecules may reflect compensatory, protective responses in CF cells, however further elucidation of their specific roles in CF biology are out of scope of the present study.

Comparison of enriched terms in both the *CF-Meta* signature and CFG genes included terms related to both mTOR signaling as well as endocytosis. Two recent papers have reported connections between CFTR and PI3K/Akt/mTOR signaling^32,33^. In particular, Reilly et al.^33^ showed that mTOR signaling pathways are over-represented in the protein interactome of ΔF508-CFTR vs. WT-CFTR, and also presented evidence that PI3K/Akt/mTOR signaling represents a novel target for rescue of ΔF508-CFTR. The endocytosis-related gene set is also of interest because ΔF508-CFTR has a shorter residence time in the plasma membrane^34^ and is more likely than WT-CFTR to be targeted for proteasomal degradation in the endoplasmic reticulum (ER).

### Findings from the *LowTmp-Meta* signature

The general consensus on low-temperature rescue of ΔF508-CFTR is that cells shift into a more “permissive” state with regard to protein folding quality control, e.g., reducing CFTR’s interaction with proteasome-targeting heat shock proteins (HSPs) such as Hsp70 and Hsp90^12,35^. Our results corroborate this idea, in that many of the genes associated with these chaperones (including *HSPA1A, HSPA1B, HSPA6, HSPA8,* and *STIP1*) were down-regulated at low temperature. However, the response to low-temperature is broad and complex^11,12,35^, and the rescue mechanism is likely to involve aspects beyond chaperone regulation. Our analysis identified a number of additional genes and pathways whose differential expression may contribute to low-temperature rescue. First, intersection with the CFG set highlighted 17 genes (Table 2) with: (1) prior evidence to affect the trafficking of ΔF508-CFTR, (2) significant differential expression with low-temperature treatment; and where (3) the direction of the transcriptional shift in relation to the functional genomics data predicts a net positive effect on the trafficking or function of ΔF508-CFTR. While this list includes a number of genes directly related to the chaperones mentioned above, it also includes less obvious genes such as *EMC8* (ER-membrane protein complex subunit 8), *RNASEL* (Ribonuclease L), and *PANK1* (pantothenate kinase 1). Also, within the enriched CF MetaMiner pathway “ΔF508-CFTR Traffic: ER-to-Golgi in CF,” we observed striking down-regulation of many genes encoding components of the COPII machinery. This finding is in line with prior work on lower-temperature (15 °C) treatment of HeLa cells, which revealed that the activity of the COPII machinery is temperature-sensitive^36^. Reduced COPII activity could lead to buildup of unfolded proteins in the ER, eventually activating the UPR. Finally, we observed elevated expression (Fig. 4, adjusted *p* < 0.02) of *HSPA5* (BiP/*GRP78*), a key component of the UPR which tends to improve ER protein folding.

We also found that genes involved in the CFTR interactome (i.e., those encoding proteins that interact with WT-CFTR, ΔF508-CFTR, or both) were significantly down-regulated in the *LowTmp-Meta* signature. This is interesting in light of the observation by^12^ that low-temperature exposure eliminates 85% of ΔF508-CFTR–specific interactions after 24 h. In fact, the WT-CFTR interactome was more down-regulated than ΔF508-CFTR–specific genes. Nevertheless, transcriptional modulation of the ΔF508-CFTR interactome may also help to explain low-temperature rescue.

Intersection of the enriched terms from the *LowTemp-Meta* and CFG analyses did not reveal a significant degree of overlap. This is likely explained by the fact that low temperature treatment induces broad effects on the cellular transcriptome which, while exerting effects on CFTR trafficking, includes additional biology, such as immune signaling, that is not specifically linked to CFTR.

### Findings from RNAi signatures

Although the RNAi interventions were identified in two related studies, we did not necessarily expect them to share a common rescue mechanism. Two of the molecules tested, miR-138 and SIN3A, are complex transcriptional regulators^4^, whereas the other two, NEDD8 and SYVN1, have more specific functions in the ubiquitin/proteasome system^7^. Because we found that their corresponding DEGs overlapped significantly, however, we applied meta-analysis to study this shared signal. The meta-analysis revealed an increase in the number of DEGs relative to the individual RNAi signatures, supporting the existence of shared biological signal.

Analysis of the *RNAi-Meta* signature revealed highly significant enrichment in PERK-mediated UPR. Evidence for UPR activity was corroborated in enrichments from the individual signatures, including those for NEDD8 and SYVN1. Inhibiting the function of these two ubiquitin/proteasome components could conceivably induce the UPR, as both factors support the proteasomal degradation of many proteins. Because evidence for UPR activation was observed not only in these signatures, but also in many other ΔF508-CFTR rescue signatures, we hypothesize that this pathway plays a mechanistic role in rescuing ΔF508-CFTR trafficking.

Of note, the transcriptional regulation of UPR genes by the RNAi interventions was subtle relative to the low-temperature treatments (see Fig. 5). This may be because the RNAi interventions induced a “low-dose” UPR activity that nonetheless yielded levels of rescue similar to those achieved by low-temperature treatment^4,7^. If it is true that low-dose UPR activation rescues ΔF508-CFTR, this may represent a more tractable therapeutic avenue than more robust interventions that induce the UPR more strongly^37^. That said, it would still be important to determine whether such a strategy would be safe and effective over prolonged exposure.

### Additional contributions

In addition to the insights provided by our analyses, other outputs of this study may yield additional value. First, the CFTR Gene Set Library, compiled from 34 publications, provides a resource for future genomic studies of CF and CFTR. The Library includes the CFG set, comprising roughly 300 genes from 25 publications that have demonstrated effects on CFTR surface expression or function. In addition, the three core signatures (Additional File 2) and Circos visualizations (Figs. 4 and 5) may facilitate insights into CF disease and rescue beyond what has been highlighted here.

### Discussion of approach: advantages and limitations

Our informatics approach has several key advantages. First, although meta-analyses of CF-associated expression data have been performed previously^14,22,38–40^, ours is the largest to date, and the first to include both disease and rescue phenotypes. Furthermore, although the power of prior studies was limited due to a binary approach that entailed simply comparing lists of DEGs, we performed quantitative meta-analyses (i.e., combining fold-change estimates across studies) to take fuller advantage of the available data. Our integrative approach revealed shared signals across multiple tissue types and interventions. Finally, while preserving the unbiased nature of whole-genome methods, we focused our analysis through the lens of CF- and CFTR-specific gene sets to take advantage of the abundant prior domain knowledge in the CF field.

Notably in this regard, we applied meta-analysis after differential expression of the individual datasets, rather than performing a single differential expression analysis with all aggregated samples. We found the former approach preferable as it avoids assumptions that the underlying data are directly comparable. Although the latter approach could be pursued by first applying batch correction to samples from individual studies, this would likely remove signal in addition to noise.

Our approach also has limitations. First, all data were obtained from bulk mRNA platforms which lack information regarding multiple splicing or cell-to-cell variability. This feature could conceal confounding effects of immune cell infiltrates in human airway brushings. Second, a general criticism applicable to any transcriptional analysis is that mRNA expression does not directly correlate with protein abundance, and thus may not capture signal relevant to the mechanism of interest (as is likely the case for C18). Accordingly, this work should be considered a means of generating hypotheses that require validation in future studies. Third, our analytic approach focused on identifying shared signal across multiple studies, based on the assumption that this signal would be more likely to be relevant to CF disease or rescue. Alternatively, one could focus on patterns that differentiate individual experiments from each other, asking, “What is different?” as opposed to “What is the same?” Although we did pursue this approach to some degree, it is possible that additional insights could be gained by committing more fully to such a strategy.

## Conclusion

This work represents, to our knowledge, the largest CF transcriptomic meta-analysis to date and presents new data characterizing chemical, genetic, and temperature-based rescue interventions. Integrative analysis across multiple experiment categories and gene sets enabled us to make richer connections than otherwise possible. This is exemplified by the multiple layers of evidence that simultaneously suggest altered activity of SGK1 and EGR1 in CF cells and also point to potential downstream effects on CFTR. Additionally, in contrast to the transcriptionally “quiet” C18 intervention, the low-temperature and RNAi-based rescue treatments converged on UPR pathways, suggesting that C18 and the other interventions act via distinct mechanisms. Moreover, this finding lends further credence to the previously noted association between UPR and ΔF508-CFTR rescue, and suggests a novel role of UPR in NEDD8-, SYVN1-, and miR-138-induced CFTR rescue.

## Methods

### Microarray experiments

#### Cell cultures

All treatments were performed in CFBE41o- cells^41^, a CF bronchial epithelial cell line that stably expresses ΔF508-CFTR. Cells were grown in submerged culture in MEM media at 37 °C for 24 h prior to treatments.

#### Temperature treatments

Temperature treatments included: (1) reduction to 27 °C for 24 h (hereafter, *LowTmp-Mc*); and (2) high-temperature (40 °C) pretreatment for 24 h prior to low-temperature (27 °C) incubation for 24 h (*HighLowTmp*). The former strategy was established in 1992 as a means of rescuing the trafficking of ΔF508-CFTR (1) and is widely used in CF research, and the latter has been shown to further increase rescue relative to low temperature alone^42^. An additional treatment using only high-temperature exposure (increase to 40 °C for 24 h, *HighTmp*) was analyzed individually and not included in the meta-analysis, based on previous observations that this condition does not rescue ΔF508-CFTR trafficking^42^. Cells subjected to each temperature intervention were compared to cells incubated for the same duration at 37 °C.

#### Chemical treatment

We also included one chemical-only intervention, 24-h incubation of CFBE41o- cells with 10 μM C18, compared with a 24-h DMSO control. C18 is a related chemical analog of the corrector compound VX-809 (Lumacaftor)^9^, which is part of a FDA approved combination therapy for treatment of individuals with the ΔF508 mutation. Both C18 and VX-809 have established rescue efficacy and are believed to interact directly with ΔF508-CFTR to improve folding stability and trafficking^8,9,43^. We used C18 instead of VX-809 due to compound availability.

#### Genetic and combination treatments

RNAi-based interventions included transfection for 24 h with siRNA sequences targeting *NEDD8*^7^, *SYVN1*^7^, or *SIN3A*^4^; or transfection with miR-138 oligonucleotides^4^. In other experiments, the same *NEDD8* or *SYVN1* knockdowns were performed in cells treated simultaneously with 10 μM C18 (*NEDD8* + *C18* and *SYVN1* + *C18*). In previous work, we showed that these combination treatments improved ΔF508-CFTR rescue relative to either intervention alone^7^. We refer to these six interventions collectively as the RNAi interventions. All RNAi interventions were compared against transfection with scrambled, negative control siRNA. Confirmation of efficacy and specificity is provided in Additional File 5.

#### RNA isolation and microarray profiling

Total RNA was isolated from cells using the RNeasy Mini Kit (Qiagen). Hybridization and scanning was performed on Illumina HumanHT-12 v4 arrays. Replicate counts and further details of these datasets and those described below are provided in Table 3. All original microarray data is included in Additional File 6 and on GEO with dataset id GSE142610.

**Table 3:** Overview of the 14 gene expression studies analyzed in the present work.

Study abbreviation	Sample source	Dataset	Platform ID	# cases, controls	# DEGs up	# DEGS down	Meta analysis group	Refs.
*CF-Bronchial*	Bronchial brushings	E-MTAB-360	GPL6098	8, 16	384	425	*CF-Meta*	^15^
*CF-Nasal*	Nasal brushings	GSE40445	GPL10097	5, 5	403	556	*CF-Meta*	^14^
*CF-WholeBlood*	Whole blood	GSE124548	GPL20301	20, 20	1036	297	*CF-Meta*	^44^
*NEDD8* + *C18*	CFBE41o- cells	GSE142610	GPL10558	4, 4	47	54	*RNAi-Meta*	–
*NEDD8*	CFBE41o- cells	GSE142610	GPL10558	5, 4	71	76	*RNAi-Meta*	–
*SYVN1* + *C18*	CFBE41o- cells	GSE142610	GPL10558	4, 4	93	129	*RNAi-Meta*	–
*SYVN1*	CFBE41o- cells	GSE142610	GPL10558	5, 4	71	108	*RNAi-Meta*	–
*MIR138*	CFBE41o- cells	GSE142610	GPL10558	4, 4	51	78	*RNAi-Meta*	–
*SIN3A*	CFBE41o- cells	GSE142610	GPL10558	4, 4	82	105	*RNAi-Meta*	–
*LowTmp-Mc*	CFBE41o- cells	GSE142610	GPL10558	5, 8	1381	1739	*LowTmp-Meta*	–
*HighLowTmp*	CFBE41o- cells	GSE142610	GPL10558	4, 8	1283	1690	*LowTmp-Meta*	–
*LowTmp-Sn*	CFBE41o- cells	GSE70442	GPL570	4, 4	1087	1293	*LowTmp-Meta*	^11^
*HighTmp*	CFBE41o- cells	GSE142610	GPL10558	4, 8	772	1027	–	–
*C18*	CFBE41o- cells	GSE142610	GPL10558	5, 4	14	20	–	–

The *Mc* and *Sn* suffixes refer to author names for disambiguation.

*Add’l* additional, *Ref* reference, *Tmp* temperature.

### Previously published microarray data

In addition to the original data described above, raw expression data were acquired from four published studies of human cells^11,14,15,44^. For CF vs. non-CF datasets, we included all publicly available datasets (as of March 2020) with *N* ≥ 3 that showed significant coherence with at least one other dataset (based on significant overlap of differentially expressed genes (DEGs) at adjusted *p* < 0.05 as described below). The first dataset (*CF-Nasal)* compared cells isolated from nasal brushings from healthy human subjects (mean age, 14.8 years) to subjects with CF (mean age, 14.0 years; homozygous ΔF508-CFTR; mean lung function, 66.5% predicted based on forced expiratory volume after one second [FEV1])^14^. Another study (*CF-Bronchial*) compared bronchial brushings from healthy human subjects (mean age, 30.3 years) to those from subjects with CF (mean age, 14.6 years; majority homozygous ΔF508-CFTR; mean lung function, 57% predicted based on FEV1)^15^. A third study (*CF-WholeBlood*) profiled whole blood from healthy human subjects (mean age, 37.9 years) as well as from subjects with CF (mean age, 21.6 years; homozygous ΔF508-CFTR; mean lung function, 74.2% predicted based on FEV1)^44^. A fourth study (*LowTmp-Sn*) compared gene expression levels in CFBE41o- cells incubated at 27 °C for 24 h versus to those in cells kept at 37 °C^11^; this comparison is analogous to our *LowTmp-Mc* experiment.

### Data processing and differential expression analysis

All microarray datasets were processed using the following pipeline. First, raw data were quantile normalized^45^ within each dataset, and then log-transformed. When appropriate, batch correction was applied using the *sva* package^46,47^. Finally, probes were mapped to Entrez IDs using Bioconductor annotation packages. Probes that mapped to multiple genes were removed, and when multiple probes mapped to the same gene, the single probe with the highest mean expression was selected. Finally, the microarray data were processed into differential expression signatures using the *Rank Products* method^48,49^. RNA-Seq data were processed end-to-end using *DESeq* with default settings from the *DESeq2* package^47,50^, which fits a generalized linear model to the raw counts based on a negative binomial distribution. For both microarray and RNA-Seq, DEGs were called at an adjusted *p* < 0.05, and |logFC| > log_2_ (1.5) (i.e. FC > 1.5). Separate DEG thresholds for the meta-analysis are described below.

### Meta-analysis

All meta-analyses were based on a standard random effects model using inverse variance pooling. More specifically, for each gene and dataset, we computed the logarithm (base 2) of the fold change (logFC) between cases and controls and the corresponding standard error of the mean (SEM). The logFC values were combined per gene using a weighted average, with weights inversely proportional to the dataset-specific SEMs. A similar approach was taken to generate an overall SEM per gene, enabling a significance estimate based on a standard *t* test. This was implemented using the *metagen* function from the *meta* R package with default parameter settings^47,51^. Multiple hypothesis correction was performed using the *qvalue* package^47,52^. DEGs were called from the resulting signatures using the following criteria: (1) adjusted *p* < 0.05; (2) |logFC| > log_2_ (1.5) in at least one study (i.e. FC > 1.5); and (3) |logFC| > log_2_ (1.3) based on the meta-analysis estimate. For the *CF-Meta* signature, genes were required to be measured in at least two of the three studies.

### Hypergeometric enrichment analysis

Hypergeometric enrichment analysis was applied to the CFG set using the *HTSanalyzeR*^47,53^ package. Gene set libraries included the following, all from MSigDB (v5): Hallmark pathways^54^ (HLMRK), Gene Ontology Biological Processes (GO BP)^55,56^, and Kyoto Encyclopedia of Genes and Genomes (KEGG) pathways^57^. In addition, we used the Kinase Enrichment Analysis (KEA) library^16^ (2013 version) downloaded from amp.pharm.mssm.edu/Enrichr/#stats and Chip-X Enrichment Analysis (ChEA) transcription factor targets^58^. We also ran enrichments against the CFTR Gene Set Library described in the Results section. Significance was defined by an adjusted *p* < 0.05.

### Gene set enrichment analysis

Gene Set Enrichment Analysis (GSEA)^59^ was applied to all core signatures using the *HTSanalyzeR*^53^ package. Gene set libraries were identical to those used in the hypergeometric enrichment analysis. Significant enrichments were defined by the following criteria: (1) adjusted *p* < 0.05; (2) FDR < 10% based on 1000 gene permutations (see *HTSanalyzeR* for details); (3) at least 2 DEGs in the gene set, and (4) ratio of observed to expected number of DEGs at least 1.5. Enrichment scores (ES) can range from − 1.0 (most extreme down-regulation) to + 1.0 (most extreme up-regulation). Note that this method takes as input the entire differential expression profile represented as all measured genes and their corresponding logFC values, avoiding the information loss that occurs when restricting a signature only to a binary set of DEGs.

### Signature correlations

All signature correlations were based on Pearson’s correlations (*r*) between gene-level logFC estimates, restricted to the set of genes measured in the corresponding pair of signatures.

## Supplementary information

Hodos MetaAnalysis_Supp Files Title Page

Additional File 1 - Individual Signatures

Additional File 2 – Core Signatures

Additional File 3A - CFTR Gene Set Library

Additional File 3B - Description of CFTR Gene Set Library

Additional File 4 - Enrichment Results

Additional File 5 - Efficacy and Specificity of RNAi Interventions

Additional File 6 - Original CFBE Microarray Data

## Data Availability

The public datasets analyzed during the current study are available in the GEO repository at www.ncbi.nlm.nih.gov/geo/query/acc.cgi?acc=GSE40445^11^, www.ncbi.nlm.nih.gov/geo/query/acc.cgi?acc=GSE124548^44^, www.ncbi.nlm.nih.gov/geo/query/acc.cgi?acc=GSE70442^14^, and the ArrayExpress repository at www.ebi.ac.uk/arrayexpress/experiments/E-MTAB-360/^15^. Original microarray data supporting the conclusions of this article are included in GEO accession GSE142610.

## Abbreviations

**Italicized gene names refer to DNA or RNA, whereas non-italicized names refer to proteins**
BP: Biological process
CF: Cystic fibrosis
CFBE: CF bronchial epithelia
CFG: CFTR functional genomics
ChEA: Chip-X enrichment analysis
DEG: Differentially expressed genes
ES: Enrichment score
FC: Fold change
FDA: Food and Drug Administration
FEV1: Forced expiratory volume at one second
GO: Gene ontology
GSEA: Gene set enrichment analysis
HLMRK: Hallmark gene set library
HSP: Heat shock protein
KEA: Kinase enrichment analysis
KEGG: Kyoto encyclopedia of genes and genomes
logFC: Logarithm (base 2) of the fold change
RNAi: RNA interference
SEM: Standard error of the mean
UPR: Unfolded protein response
WT: Wildtype
