# Supplementary material for: Integrative genomic meta-analysis reveals novel molecular insights into cystic fibrosis and ΔF508-CFTR rescue
Source: Sci Rep. 2020 Nov 25;10:20553. doi: 10.1038/s41598-020-76347-0 (PMC7689470; doi:10.1038/s41598-020-76347-0)
Supplement: Supplementary file 1 — Hodos MetaAnalysis_Supp Files Title Page [file 41598_2020_76347_MOESM1_ESM.docx]

**Integrative genomic meta-analysis reveals novel molecular insights into cystic fibrosis and ΔF508-CFTR rescue**

Rachel A. Hodos^1,2†^, Matthew D. Strub^3,4^, Shyam Ramachandran^3‡^, Li Li^1§^,

Paul B. McCray, Jr.^3,4*^, Joel T. Dudley^1*^

Additional File 1: **Individual Signatures.xlsx**

Spreadsheet containing DEGs from the fourteen differential expression signatures. PFP, percent false positive.

Additional File 2: **Core Signatures.xlsx**

Spreadsheet containing the three core signatures derived from the meta-analysis, including lists of DEGs and the entire set of measured genes, with fold-changes and *p*-values for each individual signature as well as estimates from the meta-analysis.

Additional File 3: **CFTR Gene Set Library.zip**

**Additional File 3A: CFTR Gene Set Library.xlsx**, and **Additional File 3B: Description of CFTR Gene Set Library.docx**, that contain respectively tables of all compiled gene sets, and a description and references of gene set sources.

Additional File 4: **Enrichment Results.xlsx**

Gene Set Enrichment Analysis results for all three core signatures, along with hypergeometric enrichment analysis results on the CGP gene set.

Additional File 5: **Efficacy and Specificity of RNAi Interventions.docx**

Figure and supporting text confirming efficacy and specificity of siRNA knockdown and microRNA overexpression experiments.

Additional File 6: **Original CFBE Microarray Data.xlsx**

Normalized log-transformed (base 2) fluorescence intensities reported in a gene-by-sample matrix, for all previously unpublished microarray experiments.
