# Supplementary material for: Integrative genomic meta-analysis reveals novel molecular insights into cystic fibrosis and ΔF508-CFTR rescue
Source: Sci Rep. 2020 Nov 25;10:20553. doi: 10.1038/s41598-020-76347-0 (PMC7689470; doi:10.1038/s41598-020-76347-0)
Supplement: Supplementary file 5 — Additional File 3B - Description of CFTR Gene Set Library [file 41598_2020_76347_MOESM5_ESM.docx]

**Description of CFTR Gene Set Library**

A total of 60 gene sets with relevance to CFTR were compiled. A description of these gene sets is included below. A table of all gene sets is included in Additional File 3A.

#### CFTR interactome (34 gene sets)

Proteins in the CFTR interactome, i.e. that were shown to physically interact with CFTR (either WT or ΔF508) via an immuno-precipitation based proteomic-profiling approach, were retrieved from supplementary information of Pankow et al.^1^. More specifically 638 proteins were identified in this study as the “core interactome,” i.e. high confidence interactors with either WT-CFTR or ΔF508-CFTR (or both). This list was mapped to 624 unique genes and is the CFTR “core” interactome that we refer to. These were also subdivided into all genes that interact with ΔF508-CFTR, genes that *only* interact with ΔF508-CFTR, and analogous lists for WT-CFTR. The core interactome was also subdivided into 29 different functional categories, such as “folding”, “metabolism”, and “degradation”, as annotated by the authors of the original study.

#### CFTR MetaMiner pathways (10 gene sets)

A set of nine expert-curated pathways related to CFTR processing, trafficking, and degradation were selected from the CF MetaMiner platform^2^. Examples of pathways include “Regulation of degradation of ΔF508-CFTR in CF” and “CFTR folding and maturation, normal and CF.” An additional gene set was defined from the union of genes in these pathways. These are referred to as metacore_XX

#### CFTR Functional Genomics (CFG) genes (4 gene sets)

We compiled a list of 6,188 unique genes from 25 publications^1,3-26^ that have been tested either via over-expression (OE) or siRNA KD experiments, for an effect on CFTR (either ΔF508 or WT) surface expression or function^[[1]](#footnote-1)^. Among this list are 77 genes (1.2%) with a positive effect (i.e. OE demonstrated rescue, and/or KD demonstrated reduced trafficking/function), and 236 genes (3.8%) with a negative effect (i.e. OE demonstrated reduced function and/or KD demonstrated rescue). The remaining genes were not identified to have an effect and are used as the background for statistical analyses. The four gene sets used refer to the 77 positive effectors (CFG**^+^**), 236 negative effectors (CFG**^-^**), all 294 unique effectors combined (some genes are shared between CFG**^+^** and CFG**^-^**), and the 6,188 gene background set. We believe this list is nearly comprehensive, in that we are not aware of any additional large-scale screens to identify CFTR effectors.

#### CF proteomics (4 gene sets)

In addition to the interactome data just described, we retrieved the results of a proteomics study^27^ that used mass spectrometry to measure how the expression of proteins was altered in CFBE41o- cells, in comparison to the WT counterpart HBE41o-. We used four gene lists from this study: specifically, genes whose products have higher [lower] expression in CFBE cells relative to HBEs, and genes whose products are expressed only in CFBEs [HBEs].

#### Miscellaneous (8 gene sets)

Closing out our CF gene set library are eight miscellaneous gene sets. Briefly, these include: genes that were previously identified as differentially expressed by multiple “corrector” compounds, i.e. that are known to rescue ΔF508-CFTR^24^ (including up-regulated, down-regulated, and both directions); four gene sets/pathways related to GRASP-dependent trafficking: autophagy (MSigDB ID M12441), autophagosome organization (M16504), autophagosome formation (M2537), and GRASP-dependent trafficking, manually compiled from literature^28-32^. Finally, we also included a previously compiled, literature-derived list of genes associated with CFTR trafficking from Ramachandran et al.^33^.

**REFERENCES**

1. Pankow S, Bamberger C, Calzolari D, Martínez-Bartolomé S, Lavallée-Adam M, Balch WE, Yates III JR. ∆ F508 CFTR interactome remodelling promotes rescue of cystic fibrosis*.* *Nature* 2015, 528:510-516.

2. Wright JM, Nikolsky Y, Serebryiskaya T, Wetmore DR. MetaMiner (CF): a disease-oriented bioinformatics analysis environment*.* *Protein Networks and Pathway Analysis* 2009:353-367.

3. Bomberger JM, Barnaby RL, Stanton BA. The deubiquitinating enzyme USP10 regulates the post-endocytic sorting of cystic fibrosis transmembrane conductance regulator in airway epithelial cells*.* *Journal of Biological Chemistry* 2009, 284:18778-18789.

4. Caohuy H, Jozwik C, Pollard HB. Rescue of ΔF508-CFTR by the SGK1/Nedd4-2 signaling pathway*.* *Journal of Biological Chemistry* 2009, 284:25241-25253.

5. Christianson JC, Olzmann JA, Shaler TA, Sowa ME, Bennett EJ, Richter CM, Tyler RE, Greenblatt EJ, Harper JW, Kopito RR. Defining human ERAD networks through an integrative mapping strategy*.* *Nature cell biology* 2012, 14:93-105.

6. Davezac N, Tondelier D, Lipecka J, Fanen P, Demaugre F, Debski J, Dadlez M, Schrattenholz A, Cahill MA, Edelman A. Global proteomic approach unmasks involvement of keratins 8 and 18 in the delivery of cystic fibrosis transmembrane conductance regulator (CFTR)/ΔF508‐CFTR to the plasma membrane*.* *Proteomics* 2004, 4:3833-3844.

7. Grove DE, Fan C-Y, Ren HY, Cyr DM. The endoplasmic reticulum–associated Hsp40 DNAJB12 and Hsc70 cooperate to facilitate RMA1 E3–dependent degradation of nascent CFTRΔF508*.* *Molecular biology of the cell* 2011, 22:301-314.

8. Harada K, Okiyoneda T, Hashimoto Y, Ueno K, Nakamura K, Yamahira K, Sugahara T, Shuto T, Wada I, Suico MA. Calreticulin negatively regulates the cell surface expression of cystic fibrosis transmembrane conductance regulator*.* *Journal of Biological Chemistry* 2006, 281:12841-12848.

9. Hassink GC, Zhao B, Sompallae R, Altun M, Gastaldello S, Zinin NV, Masucci MG, Lindsten K. The ER‐resident ubiquitin‐specific protease 19 participates in the UPR and rescues ERAD substrates*.* *EMBO reports* 2009, 10:755-761.

10. Henderson MJ, Vij N, Zeitlin PL. Ubiquitin C-terminal hydrolase-L1 protects cystic fibrosis transmembrane conductance regulator from early stages of proteasomal degradation*.* *Journal of Biological Chemistry* 2010, 285:11314-11325.

11. Hutt DM, Herman D, Rodrigues AP, Noel S, Pilewski JM, Matteson J, Hoch B, Kellner W, Kelly JW, Schmidt A. Reduced histone deacetylase 7 activity restores function to misfolded CFTR in cystic fibrosis*.* *Nature chemical biology* 2010, 6:25-33.

12. Kerbiriou M, Le Drévo M-A, Férec C, Trouvé P. Coupling cystic fibrosis to endoplasmic reticulum stress: differential role of Grp78 and ATF6*.* *Biochimica et Biophysica Acta (BBA)-Molecular Basis of Disease* 2007, 1772:1236-1249.

13. Le Drévo M-A, Benz N, Kerbiriou M, Giroux-Metges M-A, Pennec J-P, Trouvé P, Férec C. Annexin A5 increases the cell surface expression and the chloride channel function of the ΔF508-cystic fibrosis transmembrane regulator*.* *Biochimica et Biophysica Acta (BBA)-Molecular Basis of Disease* 2008, 1782:605-614.

14. Luciani A, Villella VR, Esposito S, Brunetti-Pierri N, Medina D, Settembre C, Gavina M, Pulze L, Giardino I, Pettoello-Mantovani M. Defective CFTR induces aggresome formation and lung inflammation in cystic fibrosis through ROS-mediated autophagy inhibition*.* *Nature cell biology* 2010, 12:863.

15. Luciani A, Villella VR, Esposito S, Gavina M, Russo I, Silano M, Guido S, Pettoello-Mantovani M, Carnuccio R, Scholte B. Targeting autophagy as a novel strategy for facilitating the therapeutic action of potentiators on ΔF508 cystic fibrosis transmembrane conductance regulator*.* *Autophagy* 2012, 8:1657-1672.

16. Norez C, Noel S, Wilke M, Bijvelds M, Jorna H, Melin P, DeJonge H, Becq F. Rescue of functional delF508‐CFTR channels in cystic fibrosis epithelial cells by the α‐glucosidase inhibitor miglustat*.* *FEBS letters* 2006, 580:2081-2086.

17. Okiyoneda T, Barrière H, Bagdány M, Rabeh WM, Du K, Höhfeld J, Young JC, Lukacs GL. Peripheral protein quality control removes unfolded CFTR from the plasma membrane*.* *Science* 2010, 329:805-810.

18. Tanguy G, Drévillon L, Arous N, Hasnain A, Hinzpeter A, Fritsch J, Goossens M, Fanen P. CSN5 binds to misfolded CFTR and promotes its degradation*.* *Biochimica et Biophysica Acta (BBA)-Molecular Cell Research* 2008, 1783:1189-1199.

19. Trzcińska-Daneluti AM, Ly D, Huynh L, Jiang C, Fladd C, Rotin D. High-content Functional Screen to Identify Proteins that Correct F508del-CFTR Function*.* *Molecular & Cellular Proteomics : MCP* 2009, 8:780-790.

20. Wang B, Heath-Engel H, Zhang D, Nguyen N, Thomas DY, Hanrahan JW, Shore GC. BAP31 Interacts with Sec61 Translocons and Promotes Retrotranslocation of CFTRΔF508 via the Derlin-1 Complex*.* *Cell* 2008, 133:1080-1092.

21. Tomati V, Sondo E, Armirotti A, Caci E, Pesce E, Marini M, Gianotti A, Jeon YJ, Cilli M, Pistorio A. Genetic inhibition of the ubiquitin ligase Rnf5 attenuates phenotypes associated to F508del cystic fibrosis mutation*.* *Scientific reports* 2015, 5:12138.

22. Perkins LA, Fisher GW, Naganbabu M, Bruchez MP. High-content Surface and Total Expression siRNA Kinase Library Screen with VX-809 Treatment Reveals Synergistic Kinase Targets that Enhance F508del-CFTR Rescue*.* *Molecular pharmaceutics* 2018.

23. Tomati V, Pesce E, Caci E, Sondo E, Scudieri P, Marini M, Amato F, Castaldo G, Ravazzolo R, Galietta LJ. High-throughput screening identifies FAU protein as a regulator of mutant cystic fibrosis transmembrane conductance regulator channel*.* *Journal of Biological Chemistry* 2018, 293:1203-1217.

24. Hegde RN, Parashuraman S, Iorio F, Ciciriello F, Capuani F, Carissimo A, Carrella D, Belcastro V, Subramanian A, Bounti L. Unravelling druggable signalling networks that control F508del-CFTR proteostasis*.* *Elife* 2015, 4:e10365.

25. Ramachandran S, Osterhaus SR, Parekh KR, Jacobi AM, Behlke MA, McCray PB. SYVN1, NEDD8, and FBXO2 Regulate ΔF508-CFTR Ubiquitin-Mediated Proteasomal Degradation*.* *Journal of Biological Chemistry* 2016:jbc. M116. 754283.

26. Trzcińska-Daneluti AM, Chen A, Nguyen L, Murchie R, Jiang C, Moffat J, Pelletier L, Rotin D. RNA interference screen to identify kinases that suppress rescue of ΔF508-CFTR*.* *Molecular & Cellular Proteomics* 2015, 14:1569-1583.

27. Rauniyar N, Gupta V, Balch WE, Yates III JR. Quantitative proteomic profiling reveals differentially regulated proteins in cystic fibrosis cells*.* *Journal of proteome research* 2014, 13:4668-4675.

28. Gee HY, Noh SH, Tang BL, Kim KH, Lee MG. Rescue of ΔF508-CFTR trafficking via a GRASP-dependent unconventional secretion pathway*.* *Cell* 2011, 146:746-760.

29. Dupont N, Jiang S, Pilli M, Ornatowski W, Bhattacharya D, Deretic V. Autophagy-based unconventional secretory pathway for extracellular delivery of IL-1β*.* *The EMBO Journal* 2011, 30:4701-4711.

30. Noh SH, Gee HY, Lee MG. Autophagosome-mediated unconventional trafficking of CFTR*.* *Pancreatology*, 13:S18.

31. Piao H, Kim J, Noh SH, Kweon H-S, Kim JY, Lee MG. Sec16A is critical for both conventional and unconventional secretion of CFTR*.* *Scientific reports* 2017, 7:39887.

32. Duran JM, Anjard C, Stefan C, Loomis WF, Malhotra V. Unconventional secretion of Acb1 is mediated by autophagosomes*.* *The Journal of Cell Biology* 2010, 188:527.

33. Ramachandran S, Karp PH, Jiang P, Ostedgaard LS, Walz AE, Fisher JT, Keshavjee S, Lennox KA, Jacobi AM, Rose SD. A microRNA network regulates expression and biosynthesis of wild-type and ΔF508 mutant cystic fibrosis transmembrane conductance regulator*.* *Proceedings of the National Academy of Sciences* 2012, 109:13362-13367.

1. as measured via changes in surface expression (band C) or subsequent channel activity. [↑](#footnote-ref-1)
