# Supplementary material for: Integrative genomic meta-analysis reveals novel molecular insights into cystic fibrosis and ΔF508-CFTR rescue
Source: Sci Rep. 2020 Nov 25;10:20553. doi: 10.1038/s41598-020-76347-0 (PMC7689470; doi:10.1038/s41598-020-76347-0)
Supplement: Supplementary file 7 — Additional File 5 - Efficacy and Specificity of RNAi Interventions [file 41598_2020_76347_MOESM7_ESM.docx]

## Efficacy and Specificity of RNAi Interventions

Shown below are expression changes of the relevant transcripts (NEDD8, SIN3A, SYVN1, miR-138) following each of the six RNAi-based treatments, based on the microarray data. Also shown are selected negative control transcripts (ACTA1 encoding an actin protein, SFRS9 encoding a member of the serine and arginine rich splicing factor protein family, and GAPDH encoding a member of the glyceraldehyde-3 phosphate dehydrogenase family). As expected, the negative controls were not significantly altered, while the transcripts targeted via siRNA or overexpression were significantly altered at *p* < 0.001. All knockdowns yielded logFC of their target transcripts below -1.5, and the overexpression of miR-138 produced a logFC of 0.59.


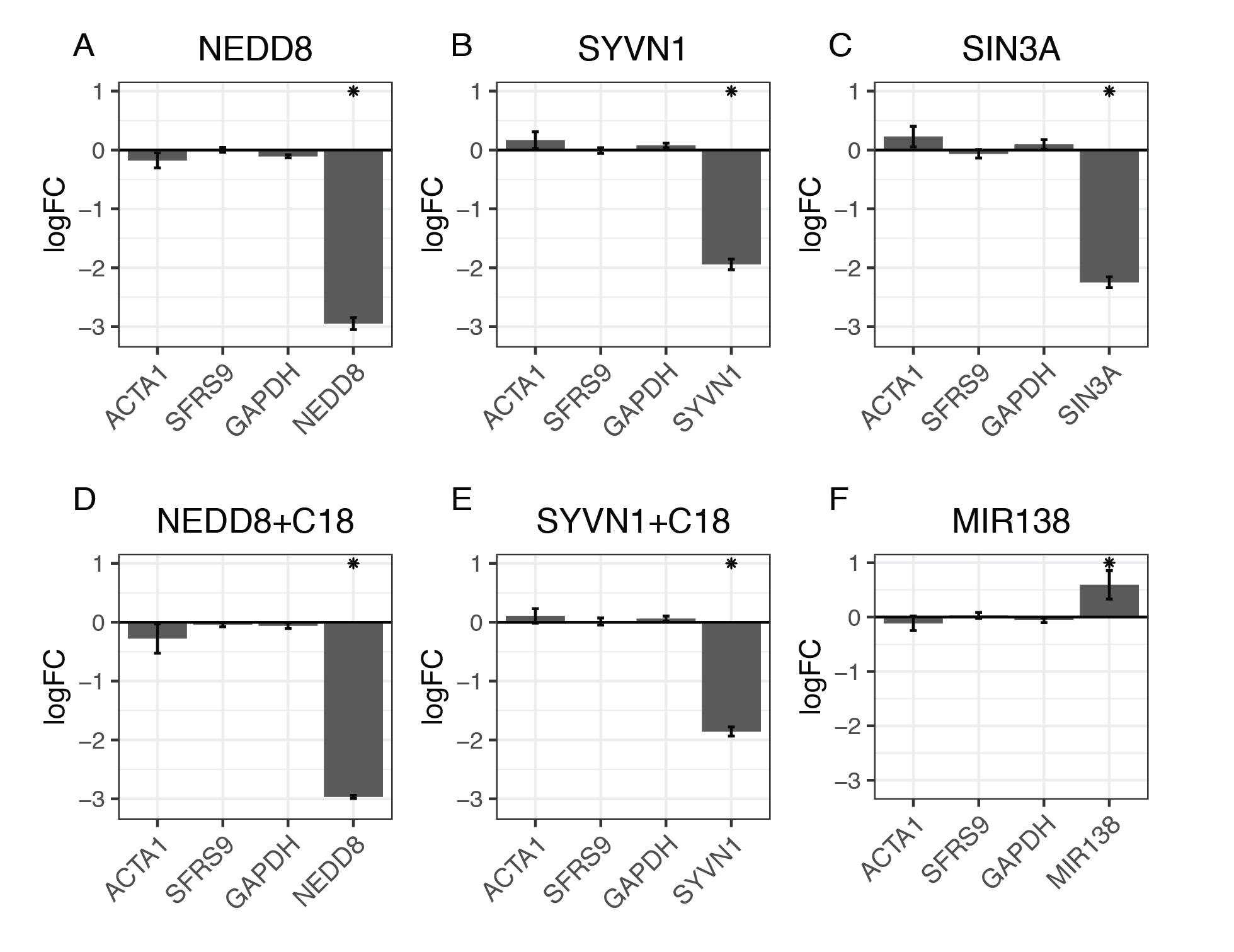


**Figure A1:** Efficacy and specificity of the six RNAi interventions in CFBE41o- cells: As indicated by the titles, the interventions are (A) NEDD8 knockdown; (B) SYVN1 knockdown; (C) SIN3A knockdown; (D) combination of NEDD8 knockdown and C18 treatment; (E) combination of SYVN1 knockdown and C18 treatment; and (F) miR-138 overexpression. Error bars indicate ±1 SEM.
